# No increased risk of Alzheimer’s disease among people with immune-mediated inflammatory diseases: findings from a longitudinal cohort study of U.S. older adults

**DOI:** 10.1186/s41927-021-00219-x

**Published:** 2021-11-12

**Authors:** Michael J. Booth, Lindsay C. Kobayashi, Mary R. Janevic, Daniel Clauw, John D. Piette

**Affiliations:** 1 Department of Health Behavior and Health Education, School of Public Health, University of Michigan, 1415 Washington Heights, Ann Arbor, MI 48130 USA; 2 Department of Epidemiology, School of Public Health, University of Michigan, Ann Arbor, MI USA; 3 Department of Anesthesiology, Rheumatology, Psychiatry, Michigan Medicine, Ann Arbor, MI USA; 4 Department of Veterans Affairs Center for Clinical Management Research, Ann Arbor, MI USA

## Abstract

**Objective:**

Immune-mediated inflammatory diseases (IMID) are characterized by systemic inflammation affecting the joints and bodily organs. Studies examining the association between individual IMIDs and the risk of Alzheimer’s disease (AD) have yielded inconsistent findings. This study examines AD risk across a group of IMIDs in a large population-based sample of older adults.

**Methods:**

Data on a national sample of US adults over age 50 was drawn from the Health and Retirement Study (HRS) and linked Medicare claims from 2006 to 2014. IMIDs include rheumatoid arthritis, psoriatic arthritis, ankylosing spondylitis, Crohn’s disease, ulcerative colitis, and related conditions. We identified IMIDs from 2006 to 2009 Medicare claims using International Classification of Diseases (ICD9-CM) codes. The date of incident AD was derived from Chronic Conditions Warehouse (CCW) identifiers. We examined the risk of AD from 2009 to 2014 using Cox proportional hazards models, both unadjusted and adjusted for age, gender, education, race, and the genetic risk factor APOE-e4.

**Results:**

One hundred seventy-one (6.02%) of the 2842 total HRS respondents with Medicare coverage and genetic data were classified with IMIDs. Over the subsequent 6 years, 9.36% of IMID patients developed AD compared to 8.57% of controls (unadjusted hazard ratio (HR): 1.09, 95% CI .66–1.81, *p* = 0.74). Adjusted HR 1.27 (95% CI 0.76–2.12, *p* = 0.35). Age (HR for 10-year increment 3.56, *p* < .001), less than high school education (HR 1.70, *p* = .007), and APOE-e4 (HR 2.61, *p* < .001 for one or two copies), were also statistically significant predictors of AD.

**Conclusion:**

HRS respondents with common IMIDs do not have increased risk of Alzheimer’s disease over a 6-year period.

## Background

Immune-mediated inflammatory disease (IMID) is a term used to describe a group of diseases that share a common inflammatory pathway wherein the immune system attacks healthy organ systems and joints [1]. Specific IMIDs vary in terms of the target of the immunopathology, for instance, the joints in rheumatoid arthritis (RA), the spine in ankylosing spondylitis (AS), the digestive tract in the inflammatory bowel diseases (IBD) Crohn’s disease and ulcerative colitis (UC), or a combination of skin and joints in psoriatic arthritis (PSA) [1, 2].

The prevalence of IMIDs in the general population is between 5 and 7% [3]. IMIDs are associated with increased mortality, disability, and poor quality of life [3]. IMIDS are also associated with increased risk of comorbid cardiovascular, renal, and infectious diseases, as well as malignancies, with lymphoma being the most common [3, 4]. Studies examining whether IMIDs are associated with an increased risk of Alzheimer’s disease (AD) generally focus on a single condition and produce mixed results [5–15].

Whether groups of IMID’s sharing a common inflammatory pathway have an increased risk of AD is unknown. AD affects approximately 5.7 million U.S. adults [16]. The impairments caused by AD may include disruption in activities of daily living, changes to behavior and personality, and an increased risk of mortality [17, 18]. In the United States, AD is the 6th leading cause of death and the 5th leading cause of death for those aged 65 and older [16]. In studies examining the association between individual IMID’s and AD or all-cause dementia, a common hypothesis is that systemic peripheral inflammation, particularly the prolonged presence of proinflammatory cytokines, may be a risk factor for dementia onset [6, 9, 11, 12]. Cytokines are signaling molecules secreted by immune cells that promote inflammation [2]. For example, in RA, tumor necrosis factor-alpha (TNF-a), interleukin-6 (IL-6), and interleukin-1b (Il-1b) are three well-studied molecules for their role in joint inflammation and are the targets of biologic Disease Modifying Anti Rheumatic Drugs (bDMARDs) used in treatment [1, 2, 19]. TNF-a is implicated in AD, and studies have examined whether TNF inhibitors reduce AD risk or progression, both in and outside of IMIDs [20–24].

Studies examining the risk of cognitive impairment in IMIDs most often focus on individual IMID diagnoses. Elevated cognitive impairment and dementia risk have been found in RA [5, 6, 8–10],, ankylosing spondylitis [12, 13], and in the inflammatory bowel diseases (IBD) Crohn’s disease and ulcerative colitis [11]. However, other studies have found no association between these conditions and AD [7, 14, 15]. Only a few studies have examined groups of autoimmune diseases and their association with AD or dementia; however, not all of the included diseases in these studies are IMIDs, and the results of the studies are mixed [9, 25, 26].

Overall, most clinic-based studies examining the risk of cognitive impairment in IMIDs have relied on small convenience samples and cross-sectional study designs. The limitations of most population-based studies are the use of administrative-based algorithms to detect IMIDs and dementia and lack of controls or matching on important sociodemographic risk factors of dementia, including education [27], gender [28], or race/ethnicity [29]. Finally, few prior studies examine the risk of AD for those with both IMID and apolipoprotein e4 (APOE-e4), a genetic variant associated with increased AD risk and earlier onset of memory loss [30]. APOE genotypes are linked to inflammation and lipid levels in RA [31], and the e4 allele may be a risk factor for PSA, Crohn’s disease, and Ulcerative colitits [32, 33]. Moreover, in a previous study, RA in midlife was associated with AD at statistical significance when adjusted for age, gender, and length of time with diagnosis, but not in a fully adjusted model that included APOE-e4 [6].

Addressing the limitations of prior studies, we examined the independent risk of AD associated with IMIDs using a large U.S. based cohort of older adults with linked medical record diagnostic data. We focused on AD risk among people with rheumatoid arthritis, psoriatic arthritis, ankylosing spondylitis, Crohn’s disease, ulcerative colitis, and related conditions, where TNF-a is implicated, and TNF-a inhibiting drugs are FDA approved for use. We further examined the role of important sociodemographic covariates of age, education, race/ethnicity, and gender, and the genetic risk factor APOE-e4. We also included a sensitivity analysis of IMID classification to better understand how classification in administrative databases affects results.

## Methods

### Data sources

The study, including access to sensitive Medicare files and genetic information, was approved by the University of Michigan Health Sciences/Behavioral Sciences Institutional Review Board (HUM00061128, HUM00152177). Data were drawn from the U.S. Health and Retirement Study (HRS), a nationally-representative longitudinal panel study of US residents 50 years of age and older [34, 35]. Approximately 20,000 participants are surveyed every 2 years, with new cohorts added every 6 years. The HRS follows participants from entry until voluntary withdrawal or death [34]. The present study sample included respondents with linkable Medicare data from 2006 to 2014, who also had linkable genetic information. Medicare and genetic data sources are discussed in more detail below.

The HRS includes information from Medicare-covered health services events for the 78–84% of respondents who authorize linkage across survey years [34]. HRS Medicare files record beneficiary level billing claims from Part A hospital insurance & Part B medical insurance covering standard provider visits from 1992 to 2016. Part C claims, also called Medicare Advantage or Medicare + Choice, are all-in-one plans offered by private companies and are not available for HRS linkage. Part D prescription drug events are available from 2006 to 2016. We limited our sample to only HRS participants with full FFS parts A & B coverage from the time they began receiving benefits, discussed in more detail in the inclusionary criteria section below.

Medicare parts A & B record the reason for a healthcare provider visit listed as International Classification of Diseases, 9th edition, Clinical Modification, or 10th edition (ICD-9-CM & ICD-10) codes, Health Care Common Procedure Coding System (HCPCS), and Current Procedural Terminology (CPT-4) codes, and contain cost-of-care, coverage, and reimbursement related information. Part D prescription events record dispensing and reimbursement related information using generic, brand name, and National Drug Codes (NDC-11).

The Health and Retirement Study genetic data is sponsored by the National Institute on Aging (grant numbersU01AG009740, RC2AG036495, and RC4AG039029) and was conducted by the University of Michigan. Genotype data are available to HRS researchers through an HRS restricted data use agreement and additional application to the database of Genotypes and Phenotypes (dbGAP) through the National Center for Biotechnology Information (NCBI). Our study used HRS Genotype Data Version1, covering 2006–2008 HRS samples. In 2006, saliva was collected using a mouthwash collection method, and in 2008, the Oragene DNA collection kit (OGR-250) [36]. Completion rates were 83% for 2006 and 84% for 2008. The NIH Center for Inherited Disease Research performed the genotyping using the Illumina Human Omni-2.5 Quad beadchip, covering approximately 2.5 million single nucleotide polymorphisms (SNPs) [36]. Further information related to specific SNP’s, quality control, and imputations of DNA variants (imputations use the 1000Genomes Project) are available at the HRS website [36].

Several genetic data products are available to HRS researchers. We used the *Candidate Genes for Cognition and Behavior* data that includes APOE files. Full documentation for these files is available [37]. We discuss our specific use of APOE isoforms in the *Covariates* section below.

### Study design

We examined the risk of AD in IMID using time-to-event analysis. Participants with and without IMID (detection of IMIDs are discussed below) were identified between 2006 and 2009, then followed from January 1st, 2009 through end of 2014. Right censoring occurred at death, the last date of a respondent’s Medicare records, or the study conclusion. We excluded prevalent AD cases at baseline in 2009 using Chronic Conditions Warehouse dates that covered the entire time respondents had Medicare coverage, also discussed in more detail below. We excluded incident IMID cases during the observation period. We chose these dates because the HRS first sampled genetic data in 2006, and 2014 was the final year of ICD-9-CM Medicare coded claims, which allows a consistent comparison of the conditions under study across all years of observation.

### Inclusionary and exclusionary criteria

Participants were eligible for inclusion in the sample if they had: 1) Full FFS parts A & B Non-HMO Medicare coverage for every year in which they had Medicare benefits, including years prior to 2006, 2) had APOE genetic data in 2006–2008, 3) were AD free at baseline on January 1, 2009, 4) had IMID at baseline or, for controls, no indication of IMID during the follow-up period, and 5) were 50 years of age or older in 2009. The final sample consisted of 2842 HRS respondents, including 171 with IMID.

### Identification of Alzheimer’s disease

The Medicare Beneficiary Summary File (BSF) contains information on beneficiaries who meet the Chronic Conditions Warehouse (CCW) criteria for various chronic conditions. The CCW criteria for AD are a single ICD9-CM diagnosis of 331.0 within a three-year observation window (meaning the beneficiary must have 3 years of FFS coverage). We discuss the strengths, limitations, and validation of this algorithm in the discussion section. We used the CCW AD dates as the date of AD incidence in the time-to-event analysis, and to create an AD free study population at baseline by excluding anyone with an AD diagnosis before January 1, 2009. Because eligible respondents were required to have full FFS coverage from the first month they began receiving Medicare benefits, we had respondents’ date of AD diagnosis occurring prior to 2006. In few instances, respondents will have first begun receiving benefits in 2006. In this scenario, they will have had 3 years of coverage from 2006 to 2009, and if an AD diagnosis occurred in this time, were excluded. However, it is possible some respondents in this scenario had an AD diagnosis prior to receiving Medicare benefits but no AD claims during the detection window. We discuss limitations related to this scenario in the discussion section.

### Identification of immune-mediated inflammatory diseases

Respondents were classified as having RA, PSA, AS, Crohn’s disease, UC, and related conditions if they had two claims-based ICD9-CM codes of 714*, 696*, 720*, 555*, and 556*, occurring at least 1 day apart, and no more than 2 years apart (Table 1). We discuss the strengths, limitations, and validation of these algorithms in the discussion section. Claims could be listed either as a principal or secondary diagnosis in Part A or Part B files. We excluded claims from non-licensed medical providers such as ambulatory services and durable medical equipment providers using the Berenson Eggers Type of Service (BETOS) indicator codes [38]. This classification method is identical to the CCW method for osteoarthritis and rheumatoid arthritis OA/RA in the BSF but includes the codes for all the IMIDs of our study. We conducted a sensitivity analysis that required three ICD9-CM codes to classify IMID using identical methods described above, only no longer requiring claims be less than 2 years apart.

**Table 1:** List of ICD9-CM codes for Immune-Mediated Inflammatory Diseases, the health and retirement study 2006–2009

ICD9-CM Code	Diagnosis
**714**	**Rheumatoid Arthritis and Other Inflammatory Polyarthropathies**
714.0	rheumatoid arthritis
714.1	felty’s syndrome
714.2	systemic rheumatoid arthritis nec
714.3	juvenile variations of inflammatory arthritis^a^
714.4	chronic postrheumatic arthropathy
714.8/.89	inflammatory polyarthropathy nec
714.81	rheumatoid lung
714.9	inflammatory polyarthropathy nos
**696**	**Psoriasis and Similar Disorders**
696.0	psoriatic arthropathy
696.1	other psoriasis
696.2	parapsoriasis
696.3/.4/.5	pityriasis rosea/ rubra pilaris/nec & nos
696.8	psoriasis related disorders nec
**720**	**Inflammatory Spondylopathies**
720.0	ankylosing spondylitis
720.1	spinal enthesopathy
720.2	sacroiliitis nec
720.8/.81	other inflammatory spondylopathy
720.89/.9	other spondylopathy nec/nos
**555**	**Regional Enteritis (Crohn’s Disease)**
555.0	regional enteritis, small intestine
555.1	regional enteritis, large intestine
555.2	regional enteritis, small/large intestine
555.9	regional enteritis nos
**556**	**Idiopathic Proctocolitis (Ulcerative Colitis)**
556.0/.1/.2/.3	ulcerative enterocolitis/ileocolitis/proctitis/prctosigmoidtis
556.4	pseudopolyposis colon
556.5/.6/.8/.9	left sided/universal/other/unspecified ulcerative colitis

*nec* not elsewhere classifiable

*nos* not otherwise specified

*ICD9-CM* International Classification of Diseases, 9th Edition, Clinical Modification

^a^Contains codes for juvenile diagnoses not found in the HRS population

### Covariates

Covariates include respondent age in 2009, gender (male/female), education (any college or more, high school or equivalent, less than high school), race (white/non-white), and a binary variable for whether a respondent has any APOE-e4 copies (no e4 copies = 0, one or two e4 copies = 1).

### Statistical methods

We examined differences in sociodemographic characteristics and the frequency of the APOE-e4 genotype between IMID and non-IMID participants using Student’s t-tests for continuous variables and Pearson’s Chi-Square test of independence for categorical variables. To examine the risk of AD associated with IMID, we plotted Kaplan Meir survival curves and calculated hazards ratios using Cox proportional hazards regression with the Breslow method for ties. We used both an unadjusted Cox regression model and a model adjusted for age, age squared, gender, education, race, and APOE-e4. We tested the global and covariate specific proportional hazards assumptions using Schoenfeld residuals in standard and log time, a likelihood ratio test comparing a model with time-covariate interactions against the nested model without time interactions, and a visual examination of log(−log (survival)) plots. We further examined the risk of AD in subsamples defined by individual diseases comprising the IMID group compared to the general population (not each other); however, small subgroup sizes precluded adjusting for covariates. We included respondents with multiple IMIDs (*n* = 9) in any of the disease-specific analyses for which they met the criteria. We conducted a sensitivity analysis requiring three IMID ICD9-CM codes, as described above.

The HRS uses a national probability sample and provides the appropriate weights for complex survey design analysis and national estimates. Our criteria requiring complete FSS linked Medicare parts A & B claims from the time respondents first received benefits and linked genetic data significantly reduced our sample from what is available in a typical HRS wave (approximately 20,000 respondents). Because of the reduction in sample size, we could not determine if our sample’s weighting reflected the original study’s probability distribution; therefore, we did not employ survey design weighting in our analysis. We performed all analyses using STATA 16.1 MP (College Station, TX).

## Results

Of the 2842 HRS respondents in the sample, 171 (6.02%) were classified as having an IMID (Table 2). Those with an IMID were on average 2 years younger than those without an IMID (mean age IMID 74.9 years, non-IMD 76.9 years, *p* = .001). Those with IMID were more often female (70.2% female for IMID, 57.9% non-IMID, *p* = .002). The IMID and non-IMID groups did not differ in the distribution of race, education, or the frequency of APOE-e4. Within the IMID group, 73 respondents were classified as RA (2.6% of total sample), 32 as PSA (1.1%), 39 as AS (1.4%), 8 as Crohn’s disease (0.3%), 10 as UC (0.4%), and 9 with two or more diagnoses (0.3%).

**Table 2:** Population characteristics of IMID and Non-IMID respondents at study index in 2009, the health and retirement study

Population Characteristic	IMID Negative (*n* = 2671)	IMID Positive (*n* = 171)	*p*
Age 2009 (mean, SD), years	76.9 (SD 7.7)	74.9 (SD 7.8)	*0.001
Sex (n, %)			*0.002
Male	1124 (42.1%)	51 (29.8%)	
Female	1547 (57.9%)	120 (70.2%)	
Race (n, %)			0.52
White/Caucasian	2396 (89.7%)	156 (91.2%)	
Non-White	275 (10.3%)	15 (8.8%)	
Education (n, %)			0.15
Less than High School	526 (19.7%)	29 (20.0%)	
High School or Equivalent	1486 (55.6%	108 (63.1%)	
Two Year College or More	659 (24.7%)	34 (19.9%)	
APOE e4 (n, %)			0.67
No Copies	2053 (76.9%)	129 (75.4%)	
One or Two copies	618 (23.1%)	42 (24.6%)	

Continuous measures tested with t-test of equal variance

Categorical measures tested using Pearson’s Chi-Square

*IMID* Immune-Mediated Inflammatory Disease

*APOE e4* Apolipoprotein e4

* Denotes statistically significant *p*-value at .05 alpha

The incident rate of AD in the IMID group was .47 per 10,000 person days and .52 per 10,000 person-days in the non-IMID group. The average follow-up time was 4.93 years. In the unadjusted Cox model for IMID as a group, the hazards ratio (HR) was 1.09 (95% CI 0.66–1.81, *p* = 0.74) (Table 3). In the adjusted model, the HR was 1.27 (0.76–2.12, *p* = 0.35). Statistically significant predictors of AD were age (HR for 10 year increase 3.56, *p* < .001), age squared (HR for 10 year increase 0.97, *p* = .006, less than high school education (HR = 1.70, *p* = 0.007), and APOE-e4 (HR 2.61, *p* < .001 for one or two copies).

**Table 3:** Hazards ratios and 95% confidence intervals of Alzheimer’s disease in IMID, the health and retirement study 2009–2014

Predictors	Unadjusted Hazards Ratio	*p*	Adjusted Hazards Ratios	*p*
IMID	1.09 (.66–1.81)	0.74	1.27 (.76–2.12)	0.35
Age (10 year increment)			3.56 (2.69–4.72)	* < .001
Age Squared (10 year increment)			.97 (.95–.99)	*.006
Gender (reference male) Female			.96 (.74–1.24)	0.73
Race (reference White) Non-White			1.4 (.92–2.13)	0.11
Education (reference two-year college or more)				
High School or Equivalent			1.24 (0.88–1.72)	0.22
Less than High School			1.70 (1.15–2.50)	*.007
APOE e4 (Reference no copies)				
One or Two copies			2.61 (2.02–3.37)	* < .001

*IMID* Immune-Mediated Inflammatory Disease

*APOE e4* Apolipoprotein e4

Hazards Ratios Calculated using Cox Proportional Hazards Models

* Denotes statistically significant *p*-value at .05 alpha

There were too few respondents with Crohn’s disease and ulcerative colitis for analysis at the individual disease level. For RA, the unadjusted HR was 1.45 (95% CI .77–2.74, *p* = 0.24); for AS the unadjusted HR was 1.14 (0.42–3.06, *p* = .80); and for PSA the unadjusted HR was .66 (0.16–2.65, *p* = .56).

**Fig. 1:**
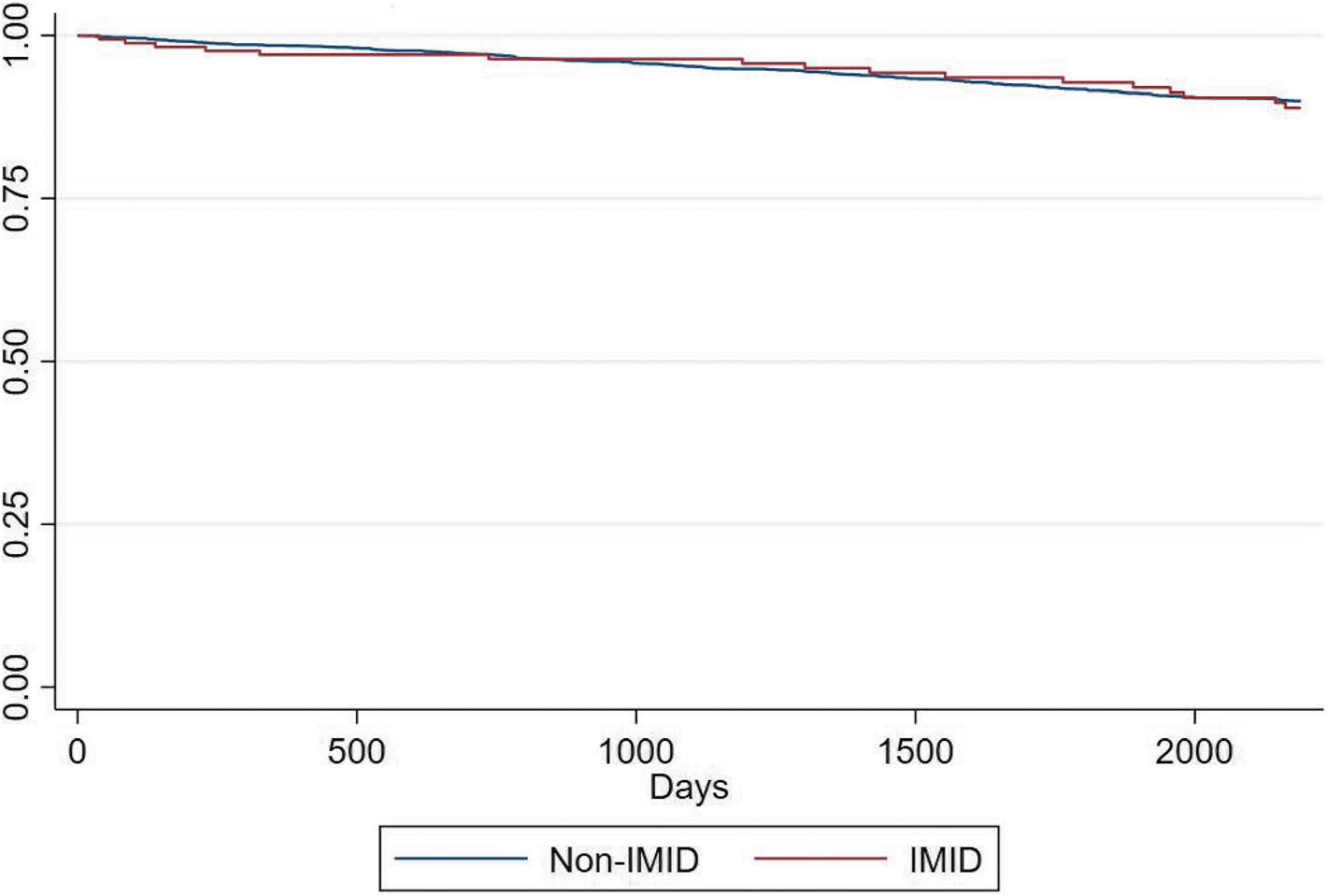
Kaplan-Meier Survival Estimates

In the sensitivity analysis identifying IMIDs using three ICD9-CM claims, there were 125 respondents in the IMID group (4.2%) out of the 2881 total respondents included. The unadjusted HR was .70 (95% CI .35–1.42, *p* = .33) and the adjusted HR .82 (.40–1.65, *p* = .57). Age, having a less than high school education, and presence of APOE-e4 were statistically significant independent predictors of AD. Kaplan-Meier survival estimates are graphed below (Fig. 1.)

## Discussion

Using a large sample of U.S. older adults, we found no difference in the risk of Alzheimer’s disease associated with immune-mediated inflammatory diseases relative to the risk in a large sample drawn from the general US population. In subgroup analyses we found no increased risk of AD associated with RA, PSA, and AS; however, we recommend cautious interpretation of disease-specific results due to small subgroup sizes. Our disease-specific models were unadjusted for important covariates due to these limitations. Our primary finding is that as a group, respondents with IMIDs had no increased risk of AD.

Many previous studies examined the risk of all-cause dementia and inflammatory autoimmune diseases, most commonly RA, whereas our study examined the risk of a specific type of dementia, Alzheimer’s disease, in a group of autoimmune diseases that included RA. Of prior studies that examined AD specifically, Wallin and colleagues found that when controlling for age, gender, and follow-up time there was an increased risk in RA; however, the association was at the borderline of statistical significance when additionally controlling for APOE-e4, smoking, and glucocorticoid/NSAID use [6]. Moreover, RA cases were identified via survey self-report, which we and several other studies have previously shown is an invalid RA measure [6, 39–42]. The use of self-reported RA classification casts doubt on the validity of these findings. Kao and colleagues found a statistically significant inverse association between RA and AD by retrospectively identifying a medical history of RA in current cases of AD [7]. This method may underestimate the RA-AD association because people with RA have an increased mortality risk and may not have survived to the point of AD measurement. Survivorship bias may explain why RA appeared to have a protective effect. Our study corrects for the shortcoming of both these studies by using a rigorous detection method for RA with two additional sensitivity measures, and by using time-to-event analysis which accounts for losses to follow-up due to death. In the case of IBD, Zhang and colleagues found an increases risk of AD in Crohn’s disease and UC, however, the study did not match participants on education and APOE-e4, two of the prominent risk factors of AD [11]. Similarly, Jang and colleagues found that AD risk is increased in AS, but likewise did not match on education or APOE-e4 [13].

In our analysis, age, education, and APOE-e4 were significant independent risk factors for AD, but not IMID’s as a group, or RA, PSA, and AS individually (with too few cases to estimate risk in Crohn’s and UC). Though in our sample, the frequency of race, education, and APOE-e4 did not differ between IMID and non-IMID groups, we chose to adjust for them based on the examination of the literature showing evidence of their associations with some IMIDs and cognitive impairment. Simulation studies show that when theoretical and empirical evidence differ for confounders in a dataset, that the theoretical confounders should still be adjusted for [43].

The strength of our study is the longitudinal design using a cohort of older adults from a nationally representative U.S. sample. An additional strength is the grouping of IMIDs that share a common inflammatory mechanism and common pharmaceutical treatments targeting this mechanism, which to our knowledge, has not been done before in research on cognitive impairment in groups of autoimmune or immune-mediated inflammatory diseases.

The most prominent limitation of our study is the use of claims-based algorithms to classify IMID and AD. The limitations of ICD-9 based algorithms come in two forms: error in coding sources and errors in validity. Coding errors may result from discrepancies between electronic and written records, miscommunication between patients and physicians, limitations in clinician’s knowledge of a specific illness, or unintentional recording errors [44]. Validity errors arise from whether or not diseases classified by an algorithm identify true cases of the disease, discussed further below.

Validity studies for the detection of specific autoimmune diseases in administrative databases are common. For example, prior research suggests that an algorithm of two ICD9-CM 714* diagnoses has a relatively low positive predictive value for detecting RA (PPV = the proportion of people identified by the algorithm with RA in the medical record) [45]. However, our study includes all of the diseases falling under 714*, not just RA. Therefore, our algorithms classify someone with RA or a related condition with any two of the 714* codes within 2 years, for example, two 714.9 codes for unspecified inflammatory polyarthropathy, or one code 714.0 for RA, and one 714.1 code for Felty’s syndrome. We did not allow counts across categories. The strength of our approach is that it includes several inflammatory autoimmune conditions in the IMID group. The limitations are that we do not know the validity of each disease-specific category. However, research shows that due to the difficulty in diagnosing many systemic autoimmune diseases, where false-positives occur in administrative data, those same subjects are often found to have a confirmed diagnosis of another related autoimmune disease [46]. Further, during the detection window in 2006–2009, the average number of claims-based diagnoses was 13.7 for RA, 7.3 for PSA, 6.4 for AS, 19.2 for Crohn’s disease, and 4.8 for UC, suggesting that those classified were receiving ongoing care. The prevalence of IMID in our sample (6.02%) is also within the estimated prevalence range of the general population [3].

In our sensitivity analysis, fewer people were detected as IMID and the HRs moved from above 1.0, to below 1.0; however, the results were statistically insignificant. The changing direction of the HRs suggests that calculation of AD risk in administrative databases is sensitive to IMID classification. Sensitivity to classification arises from validity tradeoffs depending on the strictness of a classification algorithm chosen. Less strict classifications may capture people with less severe disease who require infrequent care. At the same time, less strict criteria may include people who do not have an IMID (i.e., lower positive predictive value). More strict classification criteria in general will have higher positive predictive values, meaning a higher percentage of people detected truly have the disease, but lower sensitivity, meaning of those who truly have the disease, fewer are detected compared to less strict methods. A stricter criterion may increase the number of false negatives, moving some people who are correctly classified by a less strict algorithm to be incorrectly classified in the stricter algorithm, which may explain the changing direction of HRs in our analysis.

The same limitations present in the classification of IMIDs are present in the classification of AD. In addition to sources of coding error previously mentioned, prior research suggests that the validity of a single AD or related dementia hospital or physician-diagnosed code has 85.3% sensitivity, 94.2% specificity, 41% positive predictive value (PPV), and 99.3% negative predictive value (NPV) [47]. The relatively high sensitivity (85.3%) means most people who truly have AD will be identified as such, though the low PPV means that the algorithm identifies many people with AD who do not have it. Misclassifications of the outcome may explain our null findings compared to other studies using a different AD detection method.

Another potential limitation is the underdiagnosis of dementia in the population [48, 49]. The most prominent consideration for our results is whether under-diagnosis of dementia is differential or non-differential between our exposure and control groups. In the event there is differential classification, such that either the exposure or control group has a higher probability of under-diagnosis, than our results would be biased. We can only speculate here, however, those with IMIDs are likely in more frequent contact with clinicians than the general population, and therefore would likely encounter more opportunities for screening, referrals, and detection of dementia. If this does in fact occur, we would expect those with IMIDs to have a higher probability of dementia diagnosis, which would bias our results away from the null. We cannot say definitively how underdiagnosis differs between groups in our research and acknowledge this as a potential limitation of our results.

Another limitation is that the detection of both AD and IMIDs is limited to the timeframe in which beneficiaries have Medicare. The Medicare-restricted timeframe means some respondents could have had AD or IMID claims prior to receiving Medicare benefits, but not during our study. However, all the IMIDs in our study require ongoing care; therefore, it is unlikely someone would have an IMID prior to receiving Medicare benefits, but no indication of care thereafter. For AD, we began the study with a three-year detection window in which all respondents have complete FFS Medicare coverage meeting the CCW observation criteria for AD. We also required all respondents to have complete FFS coverage in years prior to 2006 in which they had Medicare benefits to exclude prevalent cases of AD. Because the majority of people with AD are 65 years of age and older and Medicare eligible [50], we do not believe this is a significant limitation likely to impact our results. A similar limitation is the 5-year AD detection window, which may be too short for the development of AD. However, the majority of our study population is 65 years of age or older, which has the highest incidence of AD, doubling every 5 years thereafter [51].

Residual confounding may also bias our results. Our models include covariates of age, age squared, gender, race, ethnicity, and APOE. Though we do not believe these covariates lack precision, it is possible our models do not include sufficient controls for other risk factors of AD that could affect our results. For instance, our models do not control for diabetes, cholesterol, smoking history, lifestyle factors, and other vascular risks that have been associated with AD [52]. Cardiovascular risk factors vary between different IMID’s, for instance, obesity is common in RA and PSA, but decreased in AS, UC, and Crohn’s disease [53]. Our models do not control for these possible confounders because we analyzed a group if IMIDs; accounting for variation in AD risk factors across the individual diseases of our grouping was not possible. This may result in residual confounding that should be considered when interpreting these results.”

Though we focused on a grouping of IMIDs where TNF-a is implicated, and TNF-inhibiting drugs are FDA approved for use, we did not measure TNF-a. Our results therefore do not allow us to prove or disprove an association between TNF-a and AD; we show only that in a group of IMIDs where TNF-a is implicated, there was no increased risk of AD.

Though in general the proportional hazards assumption is violated when Kaplan-Meir survival curves cross, in our case, the few number of respondents in the IMID group resulted in a blocky survival curve relative to the smooth curve in the significantly larger non-IMID group. Therefore, some visual crossing of survival curves is highly likely. We conducted extensive testing of the proportional hazards assumption and found no violations.

The benefit of using secondary data sources for a longitudinal analysis is the savings in time and cost and the lack of difficulties in study recruitment and retention common in clinic-based research. The tradeoff is a lack of validity in the diseases under study, as discussed above. We believe our research gives reason to be cautious in interpreting other studies showing an increased risk of AD in immune-mediated inflammatory diseases. We recommend future research pursue sources of validated diagnoses of IMIDs and AD to examine this relationship further. We also suggest that future research include sensitivity analyses for IMID classification when using administrative data.

## Data Availability

Restrictions apply to the availability of HRS and Medicare data, which were used for this study in a protected virtual desktop infrastructure maintained by the University of Michigan Institute for Social Research and the Health and Retirement Study. The data are not available for sharing and are accessible only through application to the Health and Retirement Study; therefore, the corresponding author will make available the Stata .do files used to manage and analyze these data upon reasonable request, which will recreate this study within the restricted Health and Retirement Study computing environment. The HRS reviewed the results of this study according to the requisite disclosure limitations and their confidentiality policy prior to publication.

## Abbreviations

AD: Alzheimer’s Disease
AHR: Adjusted Hazards Ratio
AS: Ankylosing Spondylitis
APOE-e4: Apolipoprotein epsilon four
bDMARDs: Biologic Disease-Modifying Anti-Rheumatic Drugs
CPT-4: Current Procedural Terminology codes
FFS: Fee for Service
HCPCS: Health Care Common Procedure Coding System
HR: Hazards Ratio
HRS: Health and Retirement Study
IBD: Inflammatory Bowel Disease
ICD-9-CM: International Classification of Diseases, Clinical Modification, 9th edition
ICD-10: International Classification of Diseases, 10th edition
IL-6: Interleukin-6
IL-1b: Interleukin-1b
IMID: Immune-Mediate Inflammatory Disease
PSA: Psoriatic Arthritis
PPV: Positive Predictive Value
RA: Rheumatoid Arthritis
TNF-a: Tumor Necrosis Factor Alpha
UC: Ulcerative Colitis
